# Midline Cervical Cleft: Review of an Uncommon Entity

**DOI:** 10.1155/2015/209418

**Published:** 2015-04-23

**Authors:** Liana Puscas

**Affiliations:** Division of Otolaryngology-Head and Neck Surgery, Department of Surgery, Duke University, P.O. Box 3805, Durham, NC 27710, USA

## Abstract

*Introduction*. Midline cervical cleft is a rare congenital malformation which nonetheless has a classic presentation. This study presents one of the largest single series of new patients with MCC and provides an exhaustive review and catalogue of publications from the international literature. *Materials and Methods*. Retrospective chart review performed in two academic medical centers and literature review performed with primary verification of all quoted references. *Results*. Ten patients with MCC were identified (8 boys and 2 girls). All patients presented with the classic findings of this congenital anomaly, and the length of the skin defect correlated with an increase in the patient's age. Surgical excision was complete in all cases. Thorough international literature review yielded only 195 verifiable previously reported cases. *Conclusions*. This is one of the largest series of new patients with midline cervical cleft presented in the world literature. Although rare (with less than 200 cases published to date) this entity does have a reliable presentation that should lead to rapid and accurate diagnosis. Complete surgical excision at an early age is appropriate since the anomaly increases in length commensurate with the patient's age.

## 1. Introduction

Midline cervical cleft (MCC) is a rare congenital anomaly whose embryological origin is uncertain. Review of the international literature reveals at least 195 cases reported to date (not including the 10 patients in this series). Taken together, the information from previously published case reports has shown relatively consistent anatomic and pathologic findings, but there have been few series with more than several patients, and no one has undertaken a review of all the published literature. The purpose of this study was to evaluate the physical and pathologic findings associated with this condition and to provide a comprehensive catalogue of published cases in the world's literature. The findings in this large series of ten patients illustrate the clinical and operative findings, demographics, and treatment of this unusual entity.

## 2. Materials and Methods

This study was performed as a retrospective chart review of patients treated by the University of Southern California Keck School of Medicine Department of Otolaryngology-Head and Neck Surgery and the Duke University Division of Otolaryngology-Head and Neck Surgery. Ten patients having the clinical and pathologic diagnosis of midline cervical cleft were identified. IRB approval was obtained from both USC and Duke. The charts were reviewed for history, clinical and pathologic findings as well as timing of surgical intervention and postoperative complications. Complete surgical excision of all components of the cleft was performed in every patient, and closure of the surgical wound was achieved using simple vertical closure or single versus multiple Z- or W-plasties depending on the length of the incision and the laxity of the neck tissue.

The literature search was performed by using the search terms “midline cervical cleft” and “congenital midline cervical cleft” in PubMed and then performing an exhaustive review of all possible publications (journal articles, textbook chapters, doctoral dissertations, and case reports) and references. Copies of original manuscripts were obtained from library and internet resources although it was not possible to acquire some dissertations. In these cases, utilizing email communication, librarians provided the number of new cases described in the dissertation. Whenever possible, authors were contacted directly to clarify whether or not cases had been previously reported and in some cases to provide the gender of the patients. If no information was available on a reference, the publication was not included in the table due to the inability to verify the number of new patients with MCC. Information of the gender of the patients was included whenever possible.

## 3. Results

Demographically, in this series, there were eight male and two female patients ranging in age from 2 months to 12 years. Seven children were Hispanic, two were Caucasian with Northern European lineage, and one was of Filipino descent.

**Figure 1:**
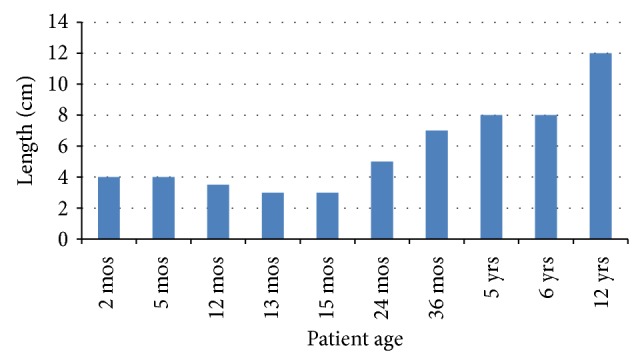
Length of involved skin relative to patient age.

Clinically, there were six consistent findings: (1) a midline, vertical atrophic skin defect, (2) a lack of adnexal elements within this skin defect, (3) a superior skin tag, (4) an inferior blind sinus, (5) a midline subcutaneous fibrous cord, and (6) an increase in the size of the defect commensurate with an increase in the patient's age. Mucous could be expressed from almost all of the patients from the inferior sinus. The length of the skin defect ranged from 3 cm to 12 cm and there was an almost direct correlation between the age of the patient and the length of the defect (Figure 1). Patients were treated with surgical excision at the time of presentation or at one year of age for those patients who presented prior to their first birthday. The fibrous cord also became more prominent as the age of the patient increased with the older patients having some restriction of neck extension. Postoperatively, one patient had a wound infection that was treated with local wound care and healed without sequelae. There were no recurrences.

Table 1 provides a catalogue of the 195 cases found in the international literature. Author, date of publication, number of cases, and gender are included as well as pertinent notes. Some authors included cases which had been presented before, and in this situation only new patients appear in the table for that publication. Overall, there were 195 cases, 77 females and 58 males and 61 instances of cases presented, but no gender was given. Cases were restricted to the accepted definition of an MCC presentation (superior skin protuberance, midline skin defect, underlying fibrous cord, and inferior blind sinus without involvement of the mandible or sternum). Cases not having at least most of these elements were not included in the final tally.

## 4. Discussion

Every patient in this series presented with the classic findings which define midline cervical cleft: a usually erythematous, vertical, and atrophic skin defect in the midline of the neck which lacks adnexal elements, a subcutaneous fibrous cord which is often longer than the overlying skin defect, a superior skin tag, and an inferior blind sinus. This constellation of clinical findings may be found clearly described in Ombredanne's work in 1949 [1]. Initially in the English literature Bailey in 1925 and Gross in 1940 called this entity “thyroglossal fistula,” but their pictures and descriptions are consistent with MCC [2, 3]. By the time he published on this subject again in 1953, Gross' nomenclature had changed to the term “midline cervical cleft” [4].

**Figure 2:**
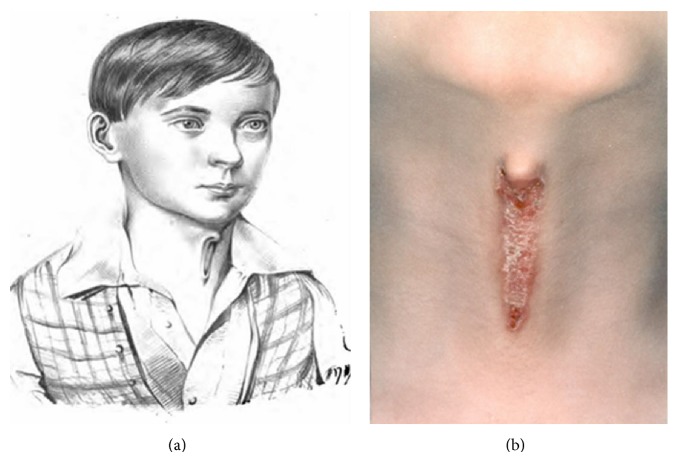
Consistency of presentation between a patient from 1848 (a) and the modern day (b).

Luschka published the first case of MCC in 1848 under the description of “Congenital Fistula of the Neck” (translated) [5]. The drawing in his report is exactly the same as a picture of one of today's patients (Figure 2). In 1864 and 1865, there appeared three reports of neck fistulas by Heusinger but one is more consistent with a bronchogenic cyst (barrel chest, cyanosis, and sinus tract that extends from the left anterior chest toward the lower neck) and the other two have fistula tracts/sinuses involving the lateral aspect of the neck [6]. Those midline cervical clefts are part of a continuum of midline defects which can be seen from two cases that were excluded from the tally: Barsky's case in 1938 in which the patient had a thick midline cord but no epithelial defect [7] and Szenes' case from 1922 in which the cleft in the neck extends inferiorly into the sternum [8].

Early reports indicated a significant preponderance of female patients [9–13], but the inclusion of the new cases presented here helps to narrow the gap between the sexes. In 61 of the cases from the world literature, the gender was not reported. This could have an effect on the gender distribution if most of these cases were found in either girls or boys. There is no apparent explanation for why girls would be affected more than boys if in fact this gender predilection is true. Most patients with MCC do not have a family history of congenital anomalies or other birth defects [11, 14], and this was true for the patients in this series as well.

MCC is considered part of the midline branchiogenic syndromes [15, 16], but it is not a true cleft in the same way as a cleft palate. Many authors have concluded that incomplete fusion of the second branchial arches is largely responsible for this entity [11, 15, 17]. Several mechanisms have been proposed to explain this incomplete fusion. The presence of amniotic adhesions and vascular anomalies may cause localized tissue ischemia, necrosis, and scarring of the developing branchial arches [18], or pressure on the developing cervical area by the closely juxtaposed pericardial roof during the fifth week of gestation may produce similar results [1]. An underlying mesodermal deficiency [9] or a disturbance in the interaction between the mesoderm and the ectoderm in the developing cervical cleft skin [10] may explain the lack of adnexal elements in the skin defect. One group of authors has proposed that the superior skin tag found in patients with this pathology is formed by a ventral outgrowth of tongue muscle [12]. Some have proposed that MCC is an inferior presentation of Tessier's facial cleft #30 which is a cleft of the mandible [19–21]. Karík included MCC under the category of branchiogenic disorders along with disorders of the mandible, tongue, lower lip, and thorax [16]. Others have proposed that MCC represents a developmental field defect [22, 23]. Bergevin et al. concluded that the surface in MCC is an invagination of endodermal cells [12] whereas Ikuzawa et al. felt that MCC formed because the median sulcus had closed insufficiently allowing a migration of aberrant multipotential cells from which the various pathologic elements of MCC are derived [24].

MCC has been associated with thyroglossal duct and branchial cysts and possibly with accessory bronchi [46, 77, 97]. Indeed, MCC must be correctly diagnosed on pathology to differentiate it from a bronchogenic cyst which can also present as a midline atrophic skin defect with a sinus tract that weeps mucous or serous fluid. Pathologically, a bronchogenic cyst has a lining of pseudostratified ciliated columnar epithelium and often has accessory tissue such as smooth muscle, seromucinous glands, and sometimes cartilage, thus resembling a very immature bronchus [121]. However, the constellation of clinical and pathologic findings can distinguish between the two entities. The erythematous, linear, and atrophic skin defect of MCC can mimic other skin disorders such as linear scleroderma, but linear scleroderma lacks the other salient characteristics of MCC [111].

Pathologic examination of the specimens from the 10 new patients presented here revealed the typical findings: the presence of skeletal muscle, minor salivary gland elements, lymphoid tissue and connective tissue, and the absence of adnexal skin elements such as hair or sebaceous glands. Sinopidis and colleagues present a very good tabular summary of the pathologic description of the individual MCC elements: the cephalic skin tag contains stratified squamous epithelium and striated muscle, the atrophic skin defect is comprised of stratified squamous epithelium without adnexal structures but with bundles of striated muscle in the dermis, and the caudal duct has squamous epithelium superficially but pseudostratified ciliated epithelium more deeply along with seromucinous glands [112].

At birth, the external layer of the cleft may consist of a weeping, red membrane which then heals to produce cicatricial skin as the patient grows. The fibrous cord, which usually extends down to the pretracheal fascia as it did in these patients, also becomes more prominent as the child grows. This is because the affected tissues lag behind in vertical growth compared with the surrounding normal neck tissue. Those patients in whom the cord is apparent even without neck extension have difficulty extending their necks. When the fibrous cord extends to the level of the mandible, a bony spur is often seen on the anterior, inferior surface of the bone secondary to the traction placed on the mandible by this tethering cord which may be severe enough to produce an open bite deformity [11, 19, 91, 102, 111].

This case series clearly demonstrates an important finding which impacts the timing of intervention in these patients. There was almost a direct correlation between the patient's age and the length of the defect (Figure 1). Whereas some previous publications recommended early excision only in those patients in whom the fibrous cord was prominent and severe producing inability to extend the neck or remodeling of the mandible [10, 11], early excision is recommended to prevent an increased scar length as well as the problems associated with a tethering midline cord [106, 111]. We treated patient surgically at the time of presentation for those over the age of one year and waited until age one for those who presented very early in life.

It is also important to completely excise the lesion. Simply transecting the fibrous cord or performing incomplete excision of the cutaneous and subcutaneous elements leads to recurrence [9–11, 74]. Closure of the surgical defect is performed with a simple vertical closure if the defect is not long and the surrounding skin is lax. The use of single or multiple W- or Z-plasties is recommended for longer defects to break up the scar and improve the cosmetic and functional results. This has long been proposed as the best way to deal with the vertical defect created by excision of the MCC and has become the usual way in which many patients are closed [40]. Early some patients with a long defect treated with a vertical closure developed neck contractures and an open bite deformity secondary to scarring after the surgery [11].

## 5. Conclusion

This case series demonstrates two interesting points. First, there was a preponderance of male patients (8/10) in contrast to previous case series in which females have predominated. Second, since the length of the defect increased as the patient's age increased, early excision of the lesion to minimize scarring is recommended. The catalogue of cases from the world literature also provides an organized list that may be helpful for future research.

## Figures and Tables

**Table 1:** Catalogue of the published world literature by author and year.

Author	Year	Number of pts	F	M	Comments
von Luschka [5]	1848	1		1	Picture of the patient in the journal is a classic MCC with superior skin tag, superficial skin defect overlying a midline cervical web
Roth [25]	1878	1		1	
Cusset [26]	1887	1	1		
Arndt [27]	1888	1		1	Articles published in 1889 and 1892 by Arndt are further discussions of the same cases but no new cases of midline cleft/sinuses
Delkeskamp [28]	1906	1		1	
Bailey [2]	1925	2	2		Called “thyroglossal fistula” but picture and description consistent with MCC
Nylander [29]	1928	2	1		
Hein [30]	1931	1	1		
Gross and Connerley [3]	1940	2	2		Called “thyroglossal fistula” but picture and description consistent with MCC
Mouchet [31]	1942	1		1	
Wynn-Williams [32]	1952	2	2		
Gross [4]	1953	4	4		It includes the 2 cases from 1940 in his total of 6; he calls them MCC now rather than thyroglossal fistula
Haym [33]	1954	1	1		
Karfik [34]	1958	2			
Maneksha [35]	1961	1	1		
Van Duyn [9]	1963	1		1	
Brown [36]	1963	2			
Schaller [37]	1963	1			
Cronin and Converse [38]	1964	1	1		Same case appears in 1990 in ch 39 Deformities of the Cervical Region in *Plastic Surgery*, vol. 3, McCarthy JG, ed. 1990, Philadelphia, WB Saunders, pp. 2078–2085
Amr [39]	1964	1		1	
Königová [40]	1965	18			Article total is 20 but 2 were already reported by Karfik in 1958; same article appears in Czech language in 1966 *Rozhl Chir* (the 1966 article's summary indicates 22 cases, but the article text indicates only 20)
Gottlieb and Lewin [10]	1966	2	2		
Monroe [41]	1966	2			Passing reference to personal experience of two patients with fissura colli medialis
Cosman and Crikelair [15]	1969	2			In discussion of the continuum of midline branchiogenic syndromes, 2 cases of MCC are mentioned
Kriens and Schuchardt [42]	1969	2	1	1	Extensive bibliography and listing of many cases until 1966
Michalland [43]	1970	6			
Sanchez Lopez Tello and Straube [44]	1970	1		1	
W. Pirsig and H. Pirsig [45]	1972	1			
French and Bale [46]	1973	1	1		
Sanchez Lopez Tello and Mueller [47]	1973	2		2	
Dargallo Reventos [48]	1975	4			In addition, one “incomplete” case is mentioned that was entirely superficial without any deeper elements and was incompletely described so it was excluded from the final tally
Ohtsuka et al. [49]	1976	1			
Post [50]	1976	2		2	
Isono et al. [51]	1977	1			
Balcells-Par and Sancho-Cerquella [52]	1977	3			
Andersen and Svendsen [53]	1978	1	1		
Minami et al. [54]	1980	1		1	
Fritzmeier and Kronsbein [55]	1982	1	1		
Wood and Deister [56]	1983	1		1	
Godbersen and Wiedemann [57]	1984	1	1		
Cotin et al. [58]	1984	1			
Lardenet et al. [59]	1984	1		1	It states 70 cases had been reported in the world literature
Gargan et al. [11]	1985	6	5	1	Article total is 12 because it includes the cases that Gross reported in 1940 and 1953
Soper et al. [60]	1986	1			
Breton et al. [61]	1987	4		4	Three of the cases are represented in another article published in 1989
Godbersen et al. [22]	1987	2	1	1	
Novák and Jakoubková [62]	1987	1		1	
Massardier [63]	1987	4			
Desnos et al. [64]	1987	4	4		
Mihara and Wada [65]	1987	1			
Lindsay et al. [66]	1988	2			
Bergevin et al. [12]	1989	1	1		
S. G. Fincher and G. G. Fincher [67]	1989	1		1	
Breton and Freidel [68]	1989				All 3 cases are included in the 1987 article, no new cases
Van der Meulen et al. [69]	1990	1		1	
Raffensperger [70]	1990	1			
van der Staak et al. [71]	1991	2	1	1	
Ikuzawa et al. [24]	1992	1	1		
Nicklaus et al. [72]	1992	2	1	1	It includes a case reported by J Friedberg in Dec 1989 in *Ped Clinc of North Amer *
Montinet et al. [73]	1992	1		1	
Maddalozzo et al. [17]	1993	5	4	1	
Liu and Lee [14]	1994	1		1	
Kececi et al. [19]	1994	1	1		Description consistent with a MCC
Maschka et al. [74]	1995	1	1		
Sfeir et al. [75]	1995	4			
Ayache et al. [76]	1997	1		1	
Andryk et al. [77]	1999	1		1	
Soderberg et al. [78]	1999	1	1		
Eastlack et al. [79]	2000	1		1	
Çetinkurşun [80]	2001	1		1	
Ercocen [23]	2002	1	1		
Genc et al. [81]	2002	1		1	
Sanchez Lopez Tello [82]	2002	1		1	Article total is 4 but includes cases of Sanchez-Lopes-Tello reported in 1970 with Straube and in 1973 with Muller
Tsukuno et al. [83]	2002	1	1		
Hirokawa et al. [84]	2003	1	1		
Joshi et al. [20]	2003	1	1		
Mylnarek et al. [85]	2003	1	1		
Daw and Patel [86]	2003	1		1	
Sannajust et al. [87]	2004	1	1		
Derbez et al. [88]	2004	5	3	2	
Bajaj et al. [89]	2004	1		1	
Tagliarini et al. [90]	2004	2	1	1	
Gardner and Moss [91]	2005	1		1	
Saha et al. [13]	2005	2	1	1	
Cochran et al. [92]	2006	1	1		
C. O. Kara and I. G. Kara [93]	2006	1		1	
Foley and Fallat [94]	2006	1			
Smith et al. [95]	2006	3	2	1	
Agag et al. [96]	2007	1		1	
Mendis and Moss [97]	2007	2	1	1	
Franzese et al. [98]	2008	2	2		
Turkyilmaz et al. [99]	2008	1			
Cheng and Gottschall [100]	2009	1	1		
Renukaswamy et al. [101]	2009	4	1	3	
Sharma et al. [102]	2009	1		1	
Vure et al. [103]	2009	1	1		
Warden and Millar [104]	2010	1	1		
Nijkamp and Rijlaarsdam [105]	2011	1	1		
Patil et al. [106]	2011	1	1		
Grynspan et al. [107]	2012	1		1	
Jakobsen et al. [108]	2012	1	1		It presents 3 cases but two involved the mandible
McInnes et al. [109]	2012	1		1	
Doddamani et al. [110]	2012	1	1		
Mendez-Gallart et al. [111]	2012	1	1		
Sinopidis et al. [112]	2012	1	1		
Tröbs et al. [113]	2012	2		2	
Martí Fajardo [114]	2012	1		1	
Lillehei and Coran [115]	2012	1	1		
Farhadi et al. [116]	2012	2	1	1	
Mirza [117]	2013	1		1	
Saha et al. [118]	2013	2	2	0	It includes two cases initially described in Saha's 2005 paper; a case of a true cleft from the mandible to the sternum was excluded from the tally
Eom et al. [119]	2014	1	1	0	First case reported in the Korean population
Crippa et al. [120]	2014	1	1	0	
Puscas (new cases)	2015	10	2	8	
TOTAL		205	79	66	
